# Modeling of the Passive State of Construction Materials in Small Modular Reactor Primary Chemistry—Effect of Dissolved Zn

**DOI:** 10.3390/ma19030456

**Published:** 2026-01-23

**Authors:** Martin Bojinov, Iva Betova, Vasil Karastoyanov

**Affiliations:** 1 Department of Physical Chemistry, University of Chemical Technology and Metallurgy, 1756 Sofia, Bulgaria; vasko_kar@uctm.edu; 2 Institute of Electrochemistry and Energy Systems, Bulgarian Academy of Sciences, 1113 Sofia, Bulgaria; i.betova@iees.bas.bg

**Keywords:** stainless steel, nickel alloy, small modular reactor, primary coolant, corrosion release, electrochemical impedance spectroscopy, Mixed-Conduction Model

## Abstract

The Mixed-Conduction Model for oxide films is used to quantitatively interpret in situ electrochemical and ex situ surface analytical results on the corrosion of AISI 316L (an internal reactor material) and Alloy 690 (a steam generator tube material) in small modular reactor primary coolant with the addition of soluble Zn. The model parameters of alloy oxidation and corrosion release are estimated with the time of exposure up to 168 h and anodic polarization potential (up to −0.25 V vs. standard hydrogen electrode) using fitting of the transfer function to experimental impedance spectra. Model parameters of individual alloy constituents are estimated by fitting of the model equations to the atomic fraction profiles of respective elements in the formed oxide obtained by Glow-Discharge Optical Emission Spectroscopy (GDOES). Conclusions on the effect of Zn addition on film growth and cation release processes in boron-free SMR coolant are drawn and future research directions are outlined.

## 1. Introduction

A number of goals, including lowering radiation exposures, maintaining the structural integrity of internals and cladding, and reducing radioactive wastes and effluents, have been reviewed as components of an ideal water chemistry program. The nuclear industry has recently become concerned about fuel performance and related environmental issues. In that context, classical water chemistry practices need to be revised and novel coolant compositions proposed to ensure the security of the plant for its entire service life. Since the 1990s, pressurized water reactors (PWRs) have been injecting soluble zinc into their primary coolant to minimize radiation fields and the likelihood of Primary Water Stress Corrosion Cracking (PWSCC) [1,2,3]. A number of studies have unequivocally demonstrated the beneficial effect of Zn in reducing ^60^Co incorporation in oxides on primary loop materials [4,5], and several in-depth investigations of the composition and structure of such oxides have emerged in the past 10–15 years [6,7,8,9,10,11,12,13,14,15,16,17,18,19,20]. Currently, Zn water chemistry is widely used in most countries that actively maintain a fleet of PWR plants [21], and Zn injection has been introduced during hot functional testing of new-generation PWR plants [22,23]. Thus, it is an established technology with clear benefits and few drawbacks that serves to minimize radiation exposure.

Small modular reactors (SMRs) are defined as nuclear fission reactors that are sufficiently compact to be built in a factory, shipped in their entirety to installation locations and then used to supply heat and power to private buildings or commercial facilities. The term SMR refers to the size, capacity, and modular construction of these reactors. IAEA defines the power capacity of SMRs to be up to 300 MWe [24]. Compared to the traditional large nuclear power plants, they display many beneficial qualities such as enhanced safety, unit cost price, flexibility, and scalability. The International Atomic Energy Agency (IAEA) lists 15 land-based light water cooled small modular reactors (SMRs) of which 14 are of the pressurized water reactor (PWR) type [24,25]. The most common PWR SMR primary side chemistry appears to be boron-free, and pH control is planned to be achieved with ammonia, LiOH, or KOH. Zinc injections are also anticipated from the hot functional testing onwards [26,27,28].

In that respect, the present paper attempts modeling of in situ and ex situ experimental data on short-term (1 week) exposures of an internal reactor material—AISI 316L stainless steel—and the most popular steam generator material, nickel Alloy 690 to boron-free SMR primary water chemistry (6 mg dm^−3^ KOH + 50 µg dm^−3^ Zn as ZnO). First, the theoretical background, the Mixed-Conduction Model (MCM), is described in some detail, emphasizing the procedures for determination of the kinetic and transport parameters for individual oxide constituents. Second, in situ electrochemical (corrosion potential vs. time, current–potential curves during anodic polarization after exposure) are presented and discussed, followed by a description of the ex situ characterization of oxides with Glow-Discharge Optical Emission Spectroscopy (GDOES). Special attention is paid to the differences in the interaction of Zn with oxide surfaces as compared to previous works in nominal PWR coolants. The dependences of the kinetic parameters on the time of exposure and polarization potential are further discussed, emphasizing the effect of the alloy material, and the values at the end of exposure are compared to those estimated from previous data measured in the absence of dissolved zinc. A similar discussion on the relevance of the parameters of individual constituents estimated by simulation of experimental depth profiles of those components follows. Finally, some conclusions are drawn from the experimental and calculation data and directions for future research are outlined.

## 2. Theoretical Background

### 2.1. A Transfer Function for the Interpretation of Impedance Spectra

**Figure 1:**
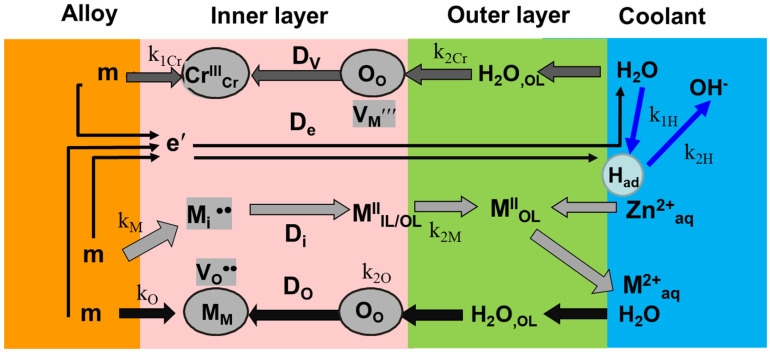
Scheme of Mixed-Conduction Model processes.

The reaction scheme illustrated in Figure 1 serves as the foundation for the transfer function obtained from the MCM in order to quantitatively replicate electro-chemical impedance data and, consequently, estimate the transport and kinetic parameters of protective layer formation and cation release [29]:

(1) $$\begin{array}{l} (M/F)\;m\overset{k_{O}}{\to }\left[M_{3}O_{4}\right]\left({4V}_{O}^{∙∙}\right)+8e^{\prime }\\ (F/S)\;\left[M_{3}O_{4}\right]\left({4V}_{O}^{∙∙}\right){+4H}_{2}O\overset{k_{2O}}{\to }M_{3}O_{4}{+8H}^{+} \end{array}$$

(2) $$\begin{array}{l} (M/F)\;m\overset{k_{M}}{\to }M_{i}^{∙∙}(\mathrm{IL})+2e^{\prime }\\ (F/S)\;3M_{i}^{∙∙}(\mathrm{IL})+4H_{2}O\overset{k_{2M}}{\to }M_{3}O_{4}(\mathrm{OL})+8H^{+}+2e^{\prime } \end{array}$$

In this sequence of reactions, m is an alloying constituent in the metallic substrate (e.g., Cr, Fe, Ni), and Kroger–Vink notation is used for the remaining symbols. Through the continuous protective layer, oxygen is transported via vacancy and cations are transported by interstitial mechanisms, coupling the interfacial steps (Figure 1).

Further, the hydrogen evolution reaction obeys the Volmer–Heyrovsky mechanism at the oxide/coolant interface (Figure 1):

(3) $$\begin{array}{l} H_{2}{O+e}^{-}\overset{k_{1H}}{\underset{k_{-1H}}{⇄}}H_{\mathrm{ad}}{(\theta )+\mathrm{OH}}^{-}\\ H_{\mathrm{ad}}{(\theta )+H}_{2}{O+e}^{-}\overset{k_{2H}}{\underset{k_{-2H}}{⇄}}H_{2}{+\mathrm{OH}}^{-} \end{array}$$

*θ* represents the coverage of the interface with hydrogen atoms (*H*_ad_) as reaction intermediates.

The total transfer function of the metal/film/solution system is derived as follows [29]:

(4) $$\begin{array}{l} Z=R_{el}+{\left(R_{F/S}^{-1}+\frac{AX}{k_{-2H}c_{H_{2}}+k_{1H}+k_{-1H}+k_{2H}+j\omega \beta }+j\omega C_{F/S}\right)}^{-1}+{\left(Z_{e}^{-1}+Z_{ion,O}^{-1}++Z_{ion,M}^{-1}\right)}^{-1}\\ R_{F/S}^{-1}=F\left(k_{-1H}b_{-1H}+k_{2H}b_{2H}\right)\bar{\theta }+F\left(k_{2H}b_{2H}c_{H_{2}}+k_{1H}b_{1H}+(y-2)k_{2M}b_{2M}\right)\left(1-\bar{\theta }\right)\\ \bar{\theta }=\frac{k_{2H}c_{H_{2}}+k_{1H}}{k_{-2H}c_{H_{2}}+k_{1H}+k_{-1H}+k_{2H}},\;A=F\left(k_{1H}+k_{-1H}-k_{-2H}c_{H_{2}}-k_{2M}-k_{2}\right)\\ X=k_{-2H}b_{-2H}c_{H_{2}}-\left(k_{-2H}b_{-2H}c_{H_{2}}-k_{1H}b_{1H}+k_{-1H}b_{-1H}-k_{2H}b_{2H}\right)\bar{\theta }-k_{1H}b_{1H}\\ Z_{e}\approx \frac{1}{2j\omega KLC_{sc}}\ln\frac{\left[1+j\omega \rho_{d}\varepsilon \varepsilon_{0}\exp\left(2KL\right)\right]}{1+j\omega \rho_{d}\varepsilon \varepsilon_{0}},\;K=\frac{F}{RT}E,\;\rho_{d}=\frac{RT}{F^{2}D_{e}}\frac{k_{2O}+k_{2M}}{k_{O}+k_{M}}\\ k_{M}=k_{M}^{0}\exp\left[\alpha_{M}\frac{F}{RT}\left\{\left(1-\alpha \right)E-EL\right\}\right],\;k_{O}=k_{O}^{0}\exp\left[\alpha_{O}\frac{F}{RT}\left\{\left(1-\alpha \right)E-EL\right\}\right],\\ k_{i}=k_{i}^{0}\exp\left(\pm b_{i}E\right),\;b_{i}=,\;i=1H,\;-1H,\;2H,\;-2H,\;2M,\;2O\\ Z_{ion,O}\approx \frac{RT}{4F^{2}k_{O}(1-\alpha )\left(1+\sqrt{1+\frac{4j\omega }{D_{O}K^{2}}}\right)},\;Z_{ion,M}\approx \frac{RT}{4F^{2}k_{M}(1-\alpha )\left(1+\sqrt{1+\frac{4j\omega }{D_{M}K^{2}}}\right)} \end{array}$$

### 2.2. Estimation of Model Parameters for Individual Oxide Constituents

#### 2.2.1. Main Processes

In addition to the oxidation path driven by oxygen ingress via the vacancy mechanism, which is represented by sequence (1), another path of oxide formation by chromium ion transport via a vacancy mechanism (Figure 1) is added in order to estimate the rate constants and diffusion coefficients of individual components of the oxide [29].

(5) $${(F/S)\;4H}_{2}O\overset{k_{2Cr}}{\to }\left[M_{3}O_{4}\right]\frac{8}{3}\left(V_{\mathrm{Cr}}^{\prime \prime \prime }\right){+8H}^{+}$$

(6) $$\left(M/F\right)\frac{8}{3}m+\frac{8}{3}\left[M_{3}O_{4}\right]\left(V_{\mathrm{Cr}}^{\prime \prime \prime }\right)\overset{k_{1\mathrm{Cr}}}{\to }\frac{8}{3}\left[M_{3}O_{4}\right]{+8e^{\prime }+V}_{m}$$

*V_m_* is a neutral vacancy in the alloy substrate. The remaining alloying elements (Fe, Ni) are transported through the oxide as interstitial cations and are released to the coolant following sequence (2).

Incorporation of Zn is assumed to proceed via emptying and filling of cation interstices [6] by the following sequence (Figure 1)

(7) $$M_{i}^{∙∙}\to M_{aq}^{2+}+V_{i}$$

(ejection of Fe or Ni resulting in an empty interstice)

(8) $$V_{i}+Zn_{aq}^{2+}\to Zn_{i}^{∙∙}$$

(incorporation of a zinc cation from the coolant in that interstice).

#### 2.2.2. Composition of the Inner Layer

The proposed calculation procedure to predict the inner and outer layer composition, as well as that of the transition region between the bulk alloy and the protective layer, is described in detail in previous work [29]; thus, only a brief summary is provided here. The flux of point defects, i.e., and oxygen vacancies *j* (*j* = *I*, interstitial cations, *V* cation, and *O* oxygen vacancies) is described by the low-field limit of the Fromhold–Cook (F-C) equation [30]:

(9) $$J_{j}(x,t)=-D_{j}\frac{\partial c_{i}(x,t)}{\partial x}-\frac{XFE}{RT}D_{j}c_{i}(x,t)$$

In this equation, *D_j_* is the diffusion coefficient of a point defect and ***E*** is the field strength in the barrier layer. The concentration of component *i* (denoted as *c_i_*) is expressed as an atomic fraction *y*_i_ = *c*_I_*V*_m,MO_ with *V*_m,MO_ the molar volume of the oxide (45 cm^3^ mol^−1^ for the oxide on stainless steel and 29 cm^3^ mol^−1^ for that on Alloy 690). For a particular cation (Fe, Ni, Cr, Zn), the corresponding F-C equations read as follows:

(10) $$\begin{array}{l} \frac{\partial y_{\mathrm{Fe}}}{\partial t}=D_{O}\frac{\partial^{2}y_{\mathrm{Fe}}}{\partial x^{2}}+\frac{2FED_{O}}{RT}\frac{\partial y_{\mathrm{Fe}}}{\partial x},\;\frac{\partial y_{\mathrm{Cr}}}{\partial t}=D_{V}\frac{\partial^{2}y_{\mathrm{Cr}}}{\partial x^{2}}+\frac{3FED_{V}}{RT}\frac{\partial y_{\mathrm{Cr}}}{\partial x},\\ \frac{\partial y_{\mathrm{Ni}}}{\partial t}=D_{O}\frac{\partial^{2}y_{\mathrm{Ni}}}{\partial x^{2}}+\frac{2FED_{O}}{RT}\frac{\partial y_{\mathrm{Ni}}}{\partial x},\;\frac{\partial y_{Zn}}{\partial t}=D_{Zn}\frac{\partial^{2}y_{Zn}}{\partial x^{2}}-\frac{2FED_{Zn}}{RT}\frac{\partial y_{Zn}}{\partial x} \end{array}$$

Bearing in mind that Zn interstitials are transported against the field. Element concentrations at the M/F interface are used as initial and inner interface boundary conditions for these equations. Boundary conditions at the film–solution interface are obtained from the steady-state solution of diffusion–migration equations of the type of (9):

(11) $$\begin{array}{l} y_{\mathrm{Fe}}(L_{\mathrm{in}},t)=\frac{k_{1\mathrm{Fe}}y_{\mathrm{Fe},a}V_{m,\mathrm{MO}}}{k_{2O}},\;y_{\mathrm{Cr}}(L_{\mathrm{in}},t)=2-k_{2\mathrm{Cr}}V_{m,\mathrm{MO}}\left(\frac{1}{k_{1\mathrm{Cr}}y_{\mathrm{Cr},a}}+\frac{RT}{3FED_{V}}\right)\\ y_{\mathrm{Ni}}(L_{\mathrm{in}},t)=\frac{k_{1\mathrm{Ni}}y_{\mathrm{Ni},a}V_{m,\mathrm{MO}}}{k_{2O}},\;y_{Zn}(L,t)=K_{enr}c_{Zn,aq}V_{m,MO} \end{array}$$

The Zn enrichment factor, representing the boundary condition for Zn at the inner layer/electrolyte interface, is the ratio of the Zn concentration there to the Zn concentration in the coolant. Thus, it can be inferred that the mechanism of Zn incorporation includes both a thermodynamic (adsorption) and a transport step [6].

The Crank–Nicolson method is employed to solve the system of Equation (10). A direct logarithmic law is adopted for the time dependence of the inner layer thickness:

(12) $$L_{\mathrm{in}}(t)=L_{\mathrm{in}}(t=0)+\frac{1}{b}\ln\left[1+V_{m,\mathrm{MO}}k_{O}be^{-bL_{in}(t=0)}t\right],\;b=\frac{3\alpha_{O}FE}{RT}$$

α_O_ is defined as the transfer coefficient of the growth reaction at the alloy/film interface.

#### 2.2.3. Composition of the Outer Oxide Layer

According to Sloppy et al. [31], the growth of the outer layer is thought to be constrained by the interstitial cation flux across the inner oxide, which is represented by the oxidation rate constant *k_i_* for an alloying element *i* (Fe, Ni, Mo). If all of the transfer coefficients for oxidation reactions are assumed equal, the time derivative of outer layer thickness is

(13) $$\frac{dL_{\mathrm{out}}(t)}{dt}=V_{m,\mathrm{MO}}(k_{1\mathrm{Fe}}y_{\mathrm{Fe},a}+k_{1\mathrm{Ni}}y_{\mathrm{Ni},a}+k_{1\mathrm{Mo}}y_{\mathrm{Mo},a})e^{-bL_{\mathrm{in}}(t)}$$

The growth law for the outer layer is obtained by integration of that equation:

(14) $$L_{\mathrm{out}}(t)=\frac{\left(k_{1\mathrm{Fe}}y_{\mathrm{Fe},a}+k_{1\mathrm{Ni}}y_{\mathrm{Ni},a}+k_{1\mathrm{Mo}}y_{\mathrm{Mo},a}\right)}{k_{O}}(L_{\mathrm{in}}(t)-L_{\mathrm{in}}(t=0))$$

Then, equations analogous to (9) are solved to calculate the cation profiles in the outer oxide:

(15) $$\begin{array}{l} \frac{\partial y_{\mathrm{Fe}}}{\partial t}=D_{I}\frac{\partial^{2}y_{\mathrm{Fe}}}{\partial x^{2}}+\frac{XF\vec{E}D_{I}}{RT}\frac{\partial y_{\mathrm{Fe}}}{\partial x},\\ \frac{\partial y_{\mathrm{Cr}}}{\partial t}=D_{I}\frac{\partial^{2}y_{\mathrm{Cr}}}{\partial x^{2}}+\frac{3F\vec{E}D_{I}}{RT}\frac{\partial y_{\mathrm{Cr}}}{\partial x},\\ \frac{\partial y_{\mathrm{Ni}}}{\partial t}=D_{I}\frac{\partial^{2}y_{\mathrm{Ni}}}{\partial x^{2}}+\frac{2F\vec{E}D_{I}}{RT}\frac{\partial y_{\mathrm{Ni}}}{\partial x},\\ \frac{\partial y_{Zn}}{\partial t}=D_{I}\frac{\partial^{2}y_{Zn}}{\partial x^{2}}-\frac{2F\vec{E}D_{I}}{RT}\frac{\partial y_{\mathrm{Zn}}}{\partial x}, \end{array}$$

with boundary conditions at the outer interface reading as

(16) $$\begin{array}{l} y_{\mathrm{Fe}}(L_{\mathrm{out}},t)=\frac{k_{1\mathrm{Fe}}y_{\mathrm{Fe},a}V_{m,\mathrm{MO}}}{k_{2\mathrm{Fe}}},y_{\mathrm{Cr}}(L_{\mathrm{out}},t)=\frac{k_{1\mathrm{Cr}}y_{\mathrm{Cr},a}}{k_{2\mathrm{Cr}}},\\ y_{\mathrm{Ni}}(L_{\mathrm{out}},t)=\frac{k_{1\mathrm{Ni}}y_{Ni,a}V_{m,\mathrm{MO}}}{k_{2\mathrm{Ni}}},y_{Zn}(L_{\mathrm{out}},t)=K_{enr,out}c_{Zn,aq}V_{m,MO} \end{array}$$

where the rate constants of ejection of individual interstitial cations from the inner oxide are marked as *k*_2Fe_, *k*_32Cr_, and *k*_2Ni_.

#### 2.2.4. Transition Metallic Layer Composition

To obtain this composition, the transport of a respective component is treated as diffusion of alloying elements from the bulk material to the interface that acts as a sink [32]:

(17) $$\begin{array}{l} \frac{\partial y_{\mathrm{FeDL}}}{\partial t}=D_{\mathrm{FeDL}}\frac{\partial^{2}y_{\mathrm{FeDL}}}{\partial x^{2}},\;\frac{\partial y_{\mathrm{CrDL}}}{\partial t}=D_{\mathrm{CrDL}}\frac{\partial^{2}y_{\mathrm{CrDL}}}{\partial x^{2}},\\ \frac{\partial y_{\mathrm{NiDL}}}{\partial t}=D_{\mathrm{NiDL}}\frac{\partial^{2}y_{\mathrm{NiDL}}}{\partial x^{2}} \end{array}$$

Only the vacancy mechanism for diffusion is considered for simplicity. The component fractions at the alloy/inner oxide interface, *y_i_*_,a_, and the corresponding contents in the bulk alloy, *y_i_*_,*DL*_, serve as boundary conditions. This ensures continuity between the inner oxide layer and the diffusion layer [32]. To compute the penetration depth of the transition layer (*L_D_*), we use a thin-layer analytical solution of the diffusion equations [32]:

(18) $$L_{D}=2\sqrt{\sum_{i}y_{i\mathrm{DL}}D_{i\mathrm{DL}}t}$$

Parameterization of the model by quantitative comparison to experimental data is presented in Section 5 below.

## 3. Materials and Methods

The experimental procedures are essentially similar to those used previously for measurements of similar materials in analogous conditions without zinc injection [33]. The composition of the studied materials (both nominal and analyzed) is detailed in Table 1. Samples were pre-treated by mechanical polishing up to emery paper grade 2400 (for stainless steel) or electropolishing in a 70% H_3_PO_4_—15%H_2_SO_4_—15%CH_3_OH electrolyte for 5 min at 9.0 V/40 °C (Alloy 690). A three-electrode flow-through cell representing the hot part of a lab-assembled re-circulation loop is used for electrochemical measurements at 280 °C/9 MPa in 6 mg dm^−3^ (0.11 mmol) KOH + 50 µg dm^−3^ (0.76 µmol dm^−3^) Zn as ZnO, continuously purged with 99.999% N_2_. The counter electrode was a Pt plate, whereas a reversible hydrogen reference electrode was approximated by a Pd foil polarized with −20 µA cm^−2^ vs. another Pt. All of the potentials in the paper are presented in the standard hydrogen electrode (SHE) scale. Corrosion potential–time, current–potential, and electrochemical impedance spectra (EIS) measurements were conducted by a Compactstat 10,030 (Ivium, Eindhoven, The Netherlands). EIS were measured from 10 kHz to 0.1 mHz (7 points per decade). A zero dc current mode featuring an ac current amplitude of 10 µA (rms) was employed, corresponding to an ac voltage of 40–50 mV (rms). Measurement of spectra with amplitudes from 1 to 10 µA checked the linearity of the data, whereas to verify causality by a Kramers–Kronig transform of the data, the measurement model [34] was applied. All of the experiments were at least triplicated to ensure reproducibility better than ±1% by magnitude and ±2° by phase shift of the impedance in the whole frequency interval studied.

Atomic fractions of constituent elements in oxides as a function of depth were estimated by a GDA 750HR instrument (Spectruma Analytik, Hof, Germany) with conditions similar to those in our previous paper [33].

## 4. Results

### 4.1. Chronopotentiometric and Voltammetric Measurements

**Figure 2:**
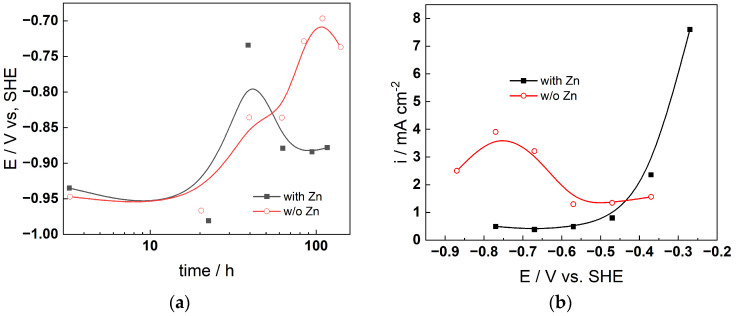
Corrosion potential vs. time (**a**) and anodic current vs. potential curve (**b**) for AISI 316L at 280 °C in primary SMR coolant (6 mg dm^−3^ KOH) with and without the addition of 50 µg dm^−3^ soluble Zn (as ZnO).

**Figure 3:**
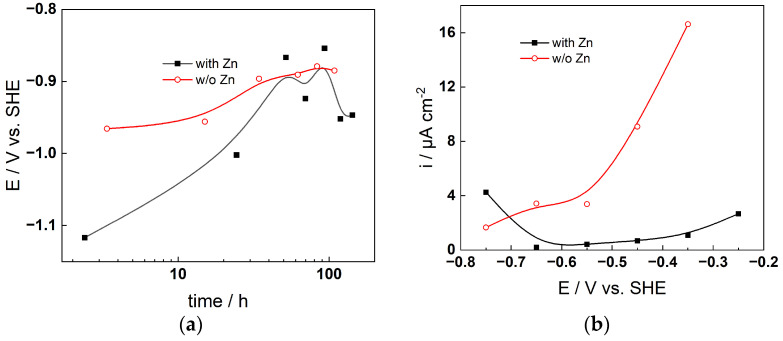
Corrosion potential vs. time (**a**) and anodic current vs. potential curve (**b**) for Alloy 690 at 280 °C in primary SMR coolant (6 mg dm^−3^ KOH) with and without the addition of 50 µg dm^−3^ soluble Zn (as ZnO).

The corrosion potential–time curves during exposure are shown in Figure 2a (AISI 316L) and Figure 3a (Alloy 690), whereas the respective anodic current–potential curves measured after 168 h of exposure are shown in Figure 2b and Figure 3b. For 316L in the presence of Zn, the corrosion potential first increases and then decreases with time, whereas it generally increases with time in the plain KOH solution. The decrease in potential at 40–50 h in the presence of Zn is most probably associated with the formation of a layer containing Zn that makes the passive state in this case different from that in plain KOH solution. Such an effect is less evident for Alloy 690, for which only the corrosion potential in the first 10–20 h is lower in the presence of Zn, and the decrease in the potential at ca. 100 h is smaller than that for AISI 316L (ca. 0.05 vs. 0.15 V).

The effect of Zn is clearly identified in the anodic current–potential curves for both materials: in the absence of Zn, transpassive oxidation starts already at −0.85 V for AISI 316L, and a shallow maximum in current is observed indicating secondary passivation. The current densities in the presence of Zn are considerably lower up to a potential of −0.4 V, after which some sort of delayed transpassive oxidation occurs (Figure 2b). For Alloy 690, transpassive oxidation starts at −0.55 V in the absence of Zn, whereas in the presence of the additive, it is not evident throughout the range of potentials investigated. Thus, it can be concluded that Zn addition alters both the passive and transpassive behavior of both studied materials, with the transpassive state being influenced more significantly by Zn for both materials regardless of the apparent differences in that process between the stainless steel and the nickel-based alloy. Since the transpassive oxidation rate is a function of the composition of the outermost layers of the passive oxide, it can be inferred that Zn significantly alters the composition and/or structure of the interface. Further proof for that is presented below.

### 4.2. Electrochemical Impedance Measurements

**Figure 4:**
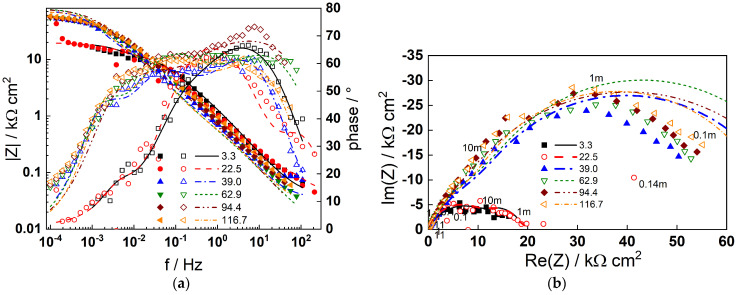
Impedance spectra of AISI 316L in 6 mg dm^−3^ KOH + 50 µg dm^−3^ dissolved Zn at 280 °C depending on time of exposure in h: (**a**) Bode plot, (**b**) complex plane plot (parameter is frequency in Hz). Points—experimental values; lines—best-fit calculation according to the model described in Section 2.

**Figure 5:**
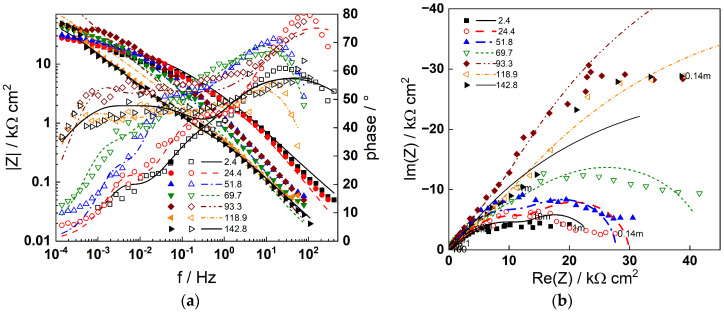
Impedance spectra of Alloy 690 in 6 mg dm^−3^ KOH + 50 µg dm^−3^ dissolved Zn at 280 °C depending on time of exposure in h: (**a**) Bode plot, (**b**) complex plane plot (parameter is frequency in Hz). Points—experimental values; lines—best-fit calculation according to the model described in Section 2.

Impedance spectra obtained at the corrosion potential are shown in Figure 4 (AISI 316L) and Figure 5 (Alloy 690) using Bode (a) and complex plane (b) representations at different exposure times. For both materials, the formation of a barrier film that slows down corrosion is indicated by an increase in the impedance magnitude at frequencies near zero (|Z|_f−0_), which is thought to be inversely proportional to the steady-state corrosion rate. Subsequently, the impedance magnitude for AISI 316L stabilizes at 40–50 h indicating a steady state with equal film formation and dissolution rates. The stabilization of impedance for Alloy 690 is observed at a later stage (ca. 100 h), in agreement with the stabilization of the corrosion potential (Figure 3a).

Four processes with different characteristic frequencies are discerned by deconvolution of the data by the distribution of relaxation times method (DRT) [35]. These reflect the electronic properties of the barrier oxide (at the highest frequencies), a two-step charge transfer process (at intermediate frequencies), and point defect transport in the barrier film (at the lowest frequency range) [33]. With the exception of the lowest frequency relaxation, all characteristic frequencies decrease with exposure time. This is particularly noticeable for Alloy 690 and is most likely related to the transition from an electropolished layer to a corrosion film. The impedance spectra calculated as a result of a complex non-linear least squares (CNLLS) fit of the data to the transfer function (Equation (4)) are shown in the figures with solid lines and demonstrate that both the magnitude and the frequency distribution of the impedance is well reproduced by the model. The dependences of the respective kinetic parameters on the time of exposure will be commented in Section 5.

**Figure 6:**
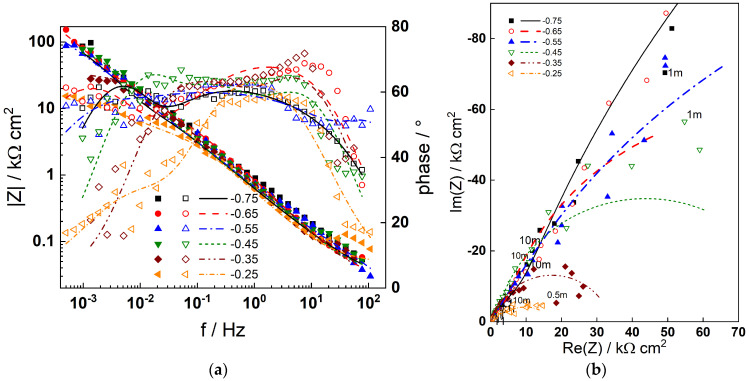
Impedance spectra of AISI 316L in 6 mg dm^−3^ KOH + 50 µg dm^−3^ dissolved Zn at 280 °C after 168 h of exposure depending on the polarization potential in V: (**a**) Bode plot, (**b**) complex plane plot (parameter is frequency in Hz). Points—experimental values; lines—best-fit calculation according to the model described in Section 2.

**Figure 7:**
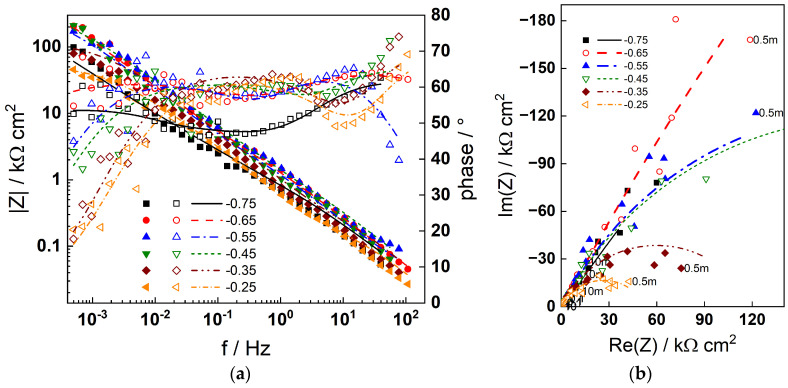
Impedance spectra of Alloy 690 in 6 mg dm^−3^ KOH + 50 µg dm^−3^ dissolved Zn at 280 °C after 168 h of exposure depending on the polarization potential in V: (**a**) Bode plot, (**b**) complex plane plot (parameter is frequency in Hz). Points—experimental values; lines—best-fit calculation according to the model described in Section 2.

The impedance spectra measured during anodic polarization after 168 h exposure of the two materials are summarized in Figure 6 (AISI 316L) and Figure 7 (Alloy 690). The number of processes detected in the spectra is similar to that at the corrosion potential, indicating that no fundamental changes in the mechanism occur; thus, the model can be used to describe the data at anodic potentials as well. This is illustrated by the fact that the model fits (lines) reproduce the experimental data (points) successfully. Once again, the physical significance of parameters depending on polarization potential will be discussed in the subsequent section.

### 4.3. Depth Profiles of Oxide Films After 168 h at the Corrosion Potential

**Figure 8:**
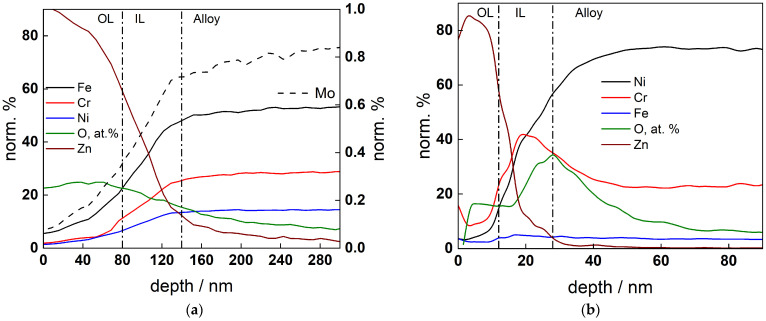
Depth profiles of normalized concentrations of metallic elements in the oxide on AISI 316L (**a**) and Alloy 690 (**b**) formed after 168 h exposure to 6 mg dm^−3^ KOH + 50 µg dm^−3^ Zn at 280 °C. Atomic concentration profile of oxygen given for comparison. The positions of the alloy/inner layer and inner layer/outer layer interface shown as vertical dash-dot lines.

Figure 8a,b illustrate the profiles of constituents in oxides formed of AISI 316L and Alloy 690 in 6 mg dm^−3^ KOH + 50 µg dm^−3^ Zn during exposure at the corrosion potential. Metallic component concentrations normalized to the total metallic element content are presented, with the atomic concentration of oxygen being given for comparison. The formed oxides exhibit a bi-layer structure, with an inner layer probably of a spinel type and an outer layer consisting essentially of a Zn-containing phase. The oxide on Alloy 690 is considerably (ca. 3 times) thinner and its inner layer is enriched in Cr, whereas this is not observed for the oxide on stainless steel. Some minor depletion of Cr is observed below the oxide, as customary for passive films on Ni alloys in high-temperature water.

The concentration of Zn in the outer layer is considerably larger than those observed in nominal PWR coolants in previous publications [4,5,6,11,12,13,14,15,16,17,18]. This is probably due to the absence of boric acid in the coolant that can lead to back dissolution of Zn and ultimately to a lower content of that metal incorporated in the oxide. The depth profile data are employed to estimate the model parameters for individual layer constituents, as emphasized in Section 5 below.

## 5. Discussion

### 5.1. Estimates of Model Parameters as a Function of Time and Applied Potential

**Figure 9:**
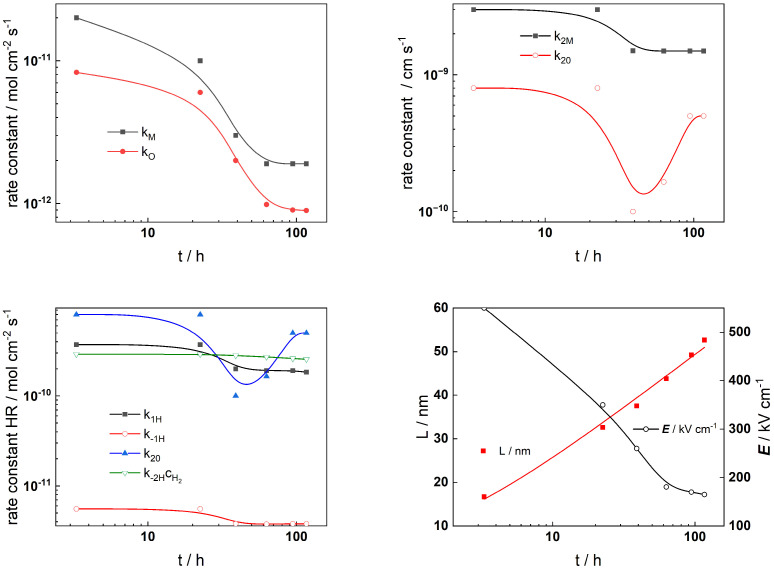
Model parameters estimated from fitting of impedance spectra to the transfer function of the model depending on time of exposure of AISI 316L to the Zn-containing SMR primary water chemistry at 280 °C for 168 h.

**Figure 10:**
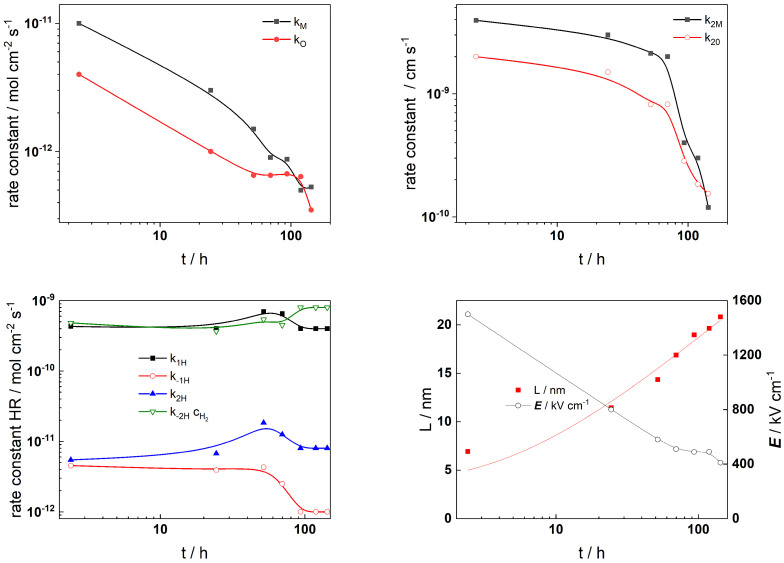
Model parameters estimated from fitting of impedance spectra to the transfer function of the model depending on time of exposure of Alloy 690 to Zn-containing SMR primary water chemistry at 280 °C for 168 h.

**Figure 11:**
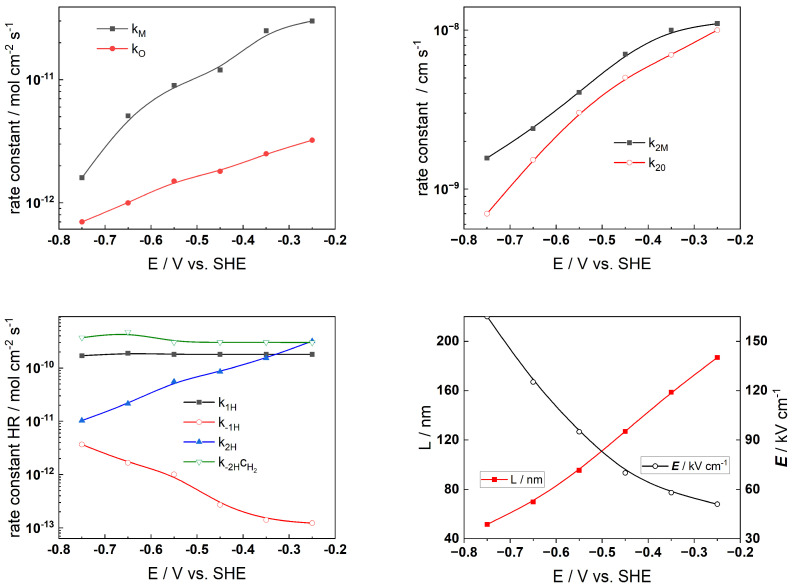
Model parameters estimated from fitting of impedance spectra to the transfer function of the model depending on applied potential after 168 h exposure of AISI 316L to Zn-containing SMR primary water chemistry.

**Figure 12:**
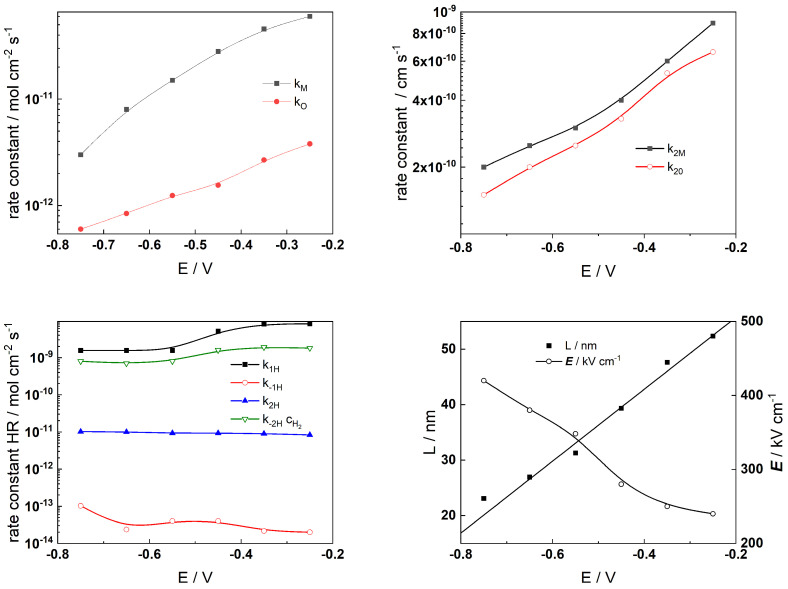
Model parameters estimated from fitting of impedance spectra to the transfer function of the model depending on applied potential after 168 h exposure of Alloy 690 to Zn-containing SMR primary water chemistry.

The Levenberg–Marquardt algorithm for CNLLS regression of the impedance data to model equations was employed to obtain parameter estimates. Several parameters were kept constant during fitting: the polarizability of the oxide/solution interface, *α* = 0.85, the oxide dielectric constant, *ε* = 25, *α*_1*H*_ = *α*_−1*H*_ = *α*_1*H*_ = *α*_−__2*H*_ = 0.5, and the steady-state concentration of dissolved H_2_, c_H2_ = 1 µmol kg^−1^ [33]. The rate constants of the interfacial reactions, the field strength, and the oxide thickness are illustrated in Figure 9 (AISI 316L) and Figure 10 (Alloy 690) vs. exposure time, whereas the corresponding dependences on applied potential during anodic polarization are shown in Figure 11 and Figure 12, respectively.

The alloy oxidation reaction rate constants (*k_M_* and *k_O_*) for both materials exhibit a power law decrease over time. Since the film thickness increases logarithmically with time, such dependences are in concert with model assumptions. The decrease becomes faster after 30–40 h of oxidation, indicating slower additional oxide growth most probably due to the slower rate of film dissolution in the presence of Zn. At the end of exposure, both rate constants tend to stabilize which indicates that a pseudo-steady state with rates of film formation and dissolution is most probably achieved. Conversely, the rate constants at the film/solution interface (*k*_2*M*_ and *k*_2*O*_) exhibit quasi-constant and comparatively large values at the beginning of exposure. Subsequently, their values change at times comparable to those at which the corrosion potential vs. time curves exhibit characteristic changes. This can be related to an alteration of the properties of the outer interface due to the formation of a Zn-rich layer that will decrease the rates of cation ejection and filling of oxygen vacancies. Such changes are also observed at similar times of exposure for the rate constants of the individual steps of the hydrogen reaction which takes place at the same interface. Thus, the formation of the Zn-rich layer has a profound influence on both the anodic and cathodic partial reactions of corrosion at the barrier film/solution interface, which has important implications for the overall oxidation and corrosion release rates. The field strength in the oxide decreases with time (or equivalently, film thickness) and reaches quasi-constant values after ca. 100 h. Space charge formation in the oxide due to significant differences in electron and ion transport rates during inner layer growth can be invoked to explain such a trend according to the following equation [36]:

(19) $$E(L)=E_{0}+L(t)\frac{F\left[2c_{O}(x)-c_{e^{\prime }}(x)-z_{i}c_{i}(x)\right]}{\varepsilon \varepsilon_{0}\left(1+\frac{L(t)}{x_{0}}\right)},\;x_{0}=\frac{\varepsilon \varepsilon_{0}RT}{{\left(2F\right)}^{2}ac_{O}(0)}$$

In this expression, the total space charge is a sum of the concentration of mobile defects (oxygen vacancies *c_O_*, electrons *c_e_*, and interstitial cations *c_i_*) and *a* is the half-jump distance for defect transport. The values of the field strength are significantly larger for the oxide on Alloy 690, indicating its more pronounced barrier properties.

Further, exponential relations with potential are followed by rate constants of interfacial reactions, as expected on electrochemical kinetic grounds. The respective transfer coefficients are low (0.1–0.2), which indicates that the configurations of initial and transition states for those processes are rather similar. The rate constants of hydrogen reactions at E = 0 do not vary with potential. One notable exception is the rate constant for hydrogen oxidation (k_−1H_), which dramatically drops as potential increases, suggesting that the mechanism of this reaction changes at higher potentials—probably due to evolution of the oxide from a corrosion film to a transpassive layer eventually containing hexavalent Cr at potentials > −0.5 V, as indicated by respective E-pH diagrams [33,36]. The oxide’s thickness grows almost linearly as the potential increases, which is consistent with the field strength’s comparatively slight (less than 30%) decline, once again related to the concentration of mobile point defects and immobile substitutional ions in the oxide. We must now recognize that the current model does not account for the transpassive oxidation reaction; as a result, the description of the processes at high anodic potentials is likely to be somewhat speculative. Further investigations of the composition and structure of the oxide at such potentials are needed to elucidate the mechanism of the process that is likely to involve the oxidation of Cr in the oxide to soluble Cr(VI) as an additional charge transfer reaction at the oxide/coolant interface, leading to increased production of cation vacancies at that interface.

The parameter values after 168 h of exposure of the studied materials at the corrosion potential are summarized in Table 2. The values estimated from experiments in Zn-free solution were taken from Refs. [33,36].

It can be concluded that the effect of Zn on the kinetics of film growth and corrosion release is significant, namely, the values of the rate constants of processes at the inner interface decrease 4–5 times in the presence of Zn, whereas those at the outer interface exhibit 2–4-times smaller values. This could be explained by the effect of Zn on the rate of dissolution of the barrier oxide that in turn affects the processes at the inner interface in order for a steady-state thickness to be preserved. Consequently, the barrier layer thickness is lower and the field strength in the oxide is larger when Zn is added to the electrolyte. The effect of Zn injection on the diffusivities of point defects in the barrier layer is somewhat smaller, but still significant (up to 2-times smaller values in the presence of Zn), which can be traced to Zn addition that promotes the formation of a less defective oxide.

### 5.2. Kinetic and Transport Parameters of Individual Oxide Constituents

**Figure 13:**
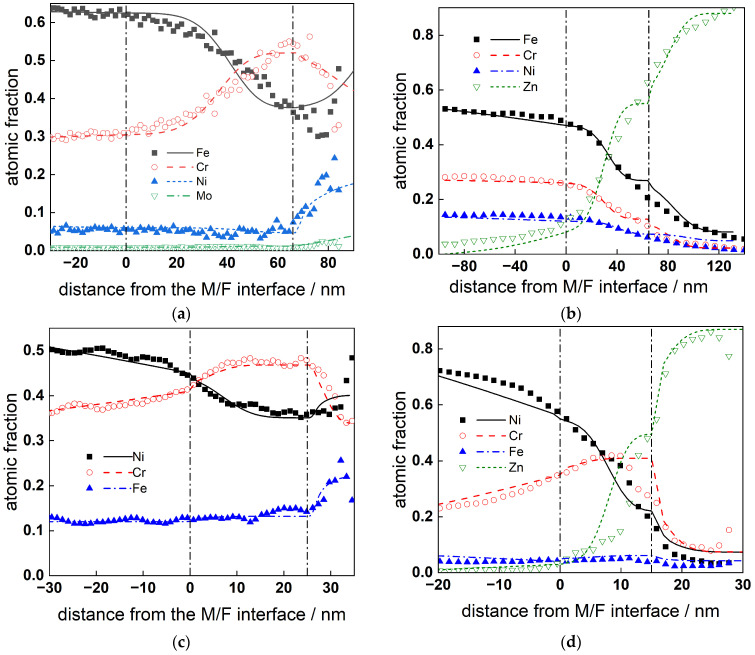
Atomic fractions of oxide constituents depending on the distance from the metal/film interface for oxides formed on AISI 316L (**a**,**b**) and Alloy 690 (**c**,**d**) in Zn-free (**a**,**c**) and Zn-containing (**b**,**d**) SMR primary water chemistry: points—experimental profiles; lines—best-fit calculation according to the proposed model.

The profiles of atomic fractions of Fe, Cr, Ni, and Zn in the oxides formed on AISI 316L and Alloy 690 for 168 h are shown in Figure 13a,b (in Zn-free coolant) and Figure 13c,d (in Zn-containing coolant) depending on the distance from the metal/film interface. The experimental profiles in Zn-free coolant are taken from Refs. [33,36]. The calculated profiles are sufficiently close to the experimental ones, indicating a good fit to the data. The respective kinetic and transport parameters depending on the studied material and the presence/absence of Zn are collected in Table 3.

From the estimated parameter values (Table 3), the effect of Zn on the reactions of oxide constituents of the oxide can also be considered significant. The thickness of the inner layer in the presence of Zn is smaller, and the composition of the outer layer is profoundly altered from a spinel layer to almost pure ZnO, which is not observed for similar materials exposed to nominal PWR coolants [8,9,10,11,12,13,14,15,16,17,18].

Additionally, the decrease in the rate constants of Fe and Ni oxidation at the inner interface is ca. 2–3-fold, with a similar decrease in the diffusivities of oxygen and cation vacancies and that of interstitial defects being somewhat smaller. The field strength in the oxide estimated from these data is comparable with that calculated from impedance spectra, and so is the thickness of the inner oxide, bearing in mind the uncertainty associated with the fact that the in-depth sensitivity of GDOES is probably larger than a monolayer.

A summary of the main findings of the present work regarding the effect of Zn addition is presented below:

- An outer layer rich in Zn (up to 80–90% of cation content) is formed on both materials during exposure. This observation differs from the usual data in nominal PWR coolants in which the Zn concentration at the interface with the coolant is usually less than 30%. A tentative explanation of this phenomenon is proposed to be the absence of boric acid that could lead to back dissolution of Zn in nominal PWR chemistries. Thus, it can be stated that the effect of Zn is more pronounced in boron-free coolants that are to be employed on the SMR primary side.
- The effect of Zn on the reactions on both metal/film and film/solution interfaces is rather significant, with the influence on the transport properties of the barrier part of oxide being also important. This also justifies the use of Zn injection in boron-free SMR primary coolants.
- The Mixed-Conduction Model is able to successfully interpret both the electrochemical data and the surface analytical results, indicating that it can also be used as a predictive tool for oxide formation and corrosion release in SMR primary coolants.

The impact of dissolved hydrogen in boron-free coolants with and without Zn addition will be the main focus of future research. These experiments are ongoing and will be published soon.

## 6. Conclusions

The present work reports the modeling of in situ electrochemical and ex situ analytical data of the corrosion and anodic oxidation mechanism of a typical reactor internal (AISI 316L) and the main steam generator tube material of classical PWRs (Alloy 690) in boron-free primary coolant with the addition of soluble Zn that is planned to be used in SMRs. By combining quantitative ex situ analysis of formed oxides with in situ electrochemical experiments, the oxidation and cation release rates of both materials in the primary coolant were estimated under conditions that closely resembled SMR operation.

Considering the industrial significance of the present study, it supports earlier experimental findings from several companies and research groups by demonstrating that a transition from B-K-Li to boron-free K primary chemistry is not detrimental to the passivity and general corrosion resistance of construction materials for prospective use in SMRs.

## Figures and Tables

**Table 1:** Nominal and GDOES-estimated compositions of the studied materials (wt.%).

	Content, wt.%	C	Fe	Cr	Cu	Mn	Ni	Mo	Si
Alloy 690	nominal	≤0.03	9.0–10.0	29.0–31.0	0.05	0.10	Bal.	0.15	0.10
	analyzed	0.025	9.1	29.5	0.03	0.07	Bal.	0.14	0.14
316L	nominal	≤0.02	Bal.	17–19	0.2–0.4	1.2–1.4	11–13	2.4–3.0	0.8
	analyzed	0.02	Bal.	17.6	0.30	1.3	12.0	2.5	0.76

**Table 2:** Kinetic and transport parameters estimated from fitting of EIS data to the transfer function of the model after 168 h exposure of the studied materials in a coolant with or without Zn addition.

Parameter	316L, *w*/*o* Zn	316L, with Zn	690, *w*/*o* Zn	690, with Zn
10^12^ *k_M_*/mol cm^−2^ s^−1^	10	1.9	2.0	0.53
10^12^ *k_O_*/mol cm^−2^ s^−1^	1.6	0.89	0.70	0.35
*L*/nm	91	52	26	21
10^8^ *D_e_*/cm^2^ s^−1^	0.50	0.64	0.56	0.93
10^9^ *k*_2*M*_/cm s^−1^	1.0	0.5	4.0	0.12
10^9^ *k*_2*O*_/cm s^−1^	1.5	0.40	2.0	0.15
10^10^ *k*_1*H*_/mol cm^−2^ s^−1^	2.0	1.8	5.5	4.0
10^12^ *k*_2*H*_/mol cm^−2^ s^−1^	5.0	3.7	7.5	4.0
10^12^ *k*_1*H*_/mol cm^−2^ s^−1^	8.0	6.0	7.5	8.0
10^4^ *k_−_*_2*H*_/cm s^−1^	2.9	2.6	4.0	4.5
*β*/nmol cm^−2^	5.0	2.0	5.0	5.0
α_O_	0.13	0.13	0.12	0.12
10^17^ *D_M_*/cm^2^ s^−1^	4.0	2.0	2.0	1.0
10^17^ *D_O_*/cm^2^ s^−1^	2.0	0.30	0.95	0.22
***E***/kV cm^−1^	92	140	340	410

**Table 3:** Kinetic and transport parameters of individual constituents of oxides produced on the studied materials with or without Zn addition to the electrolyte.

Parameter	316L, *w*/*o* Zn	316L, with Zn	690, *w*/*o* Zn	690, with Zn
10^12^ *k*_1Fe_/mol cm^−2^ s^−1^	4.0	1.5	2.0	1.3
10^12^ *k*_1Ni_/mol cm^−2^ s^−1^	2.6	1.0	1.5	1.0
10^12^ *k*_O_/mol cm^−2^ s^−1^	3.3	2.5	1.7	1.0
10^13^ *k*_3Cr_/mol cm^−2^ s^−1^	1.6	2.4	1.7	1.1
10^9^ *k*_1Cr_/cm s^−1^	4.5	4.5	3.5	3.5
10^10^ *k*_2Fe_/cm s^−1^	7.0	5.0	5.0	7.0
10^10^ *k*_2Ni_/cm s^−1^	6.0	6.0	1.0	3.0
10^18^ *D*_O_/cm^2^ s^−1^	2.85	1.0	1.25	0.90
10^18^ *D*_V_/cm^2^ s^−1^	1.90	1.0	1.20	1.1
10^18^ *D*_I_/cm^2^ s^−1^	3.0	1.5	2.0	1.2
$E/{\mathrm{kV}\;\mathrm{cm}}^{-1}$	90	120	300	350
α_O_	0.11	0.10	0.10	0.11
*L_in_*/nm	77	54	26	18

## Data Availability

The data presented in this study are available on request from the corresponding author. The data are not publicly available due to privacy restrictions.
